# Supplementary material for: Hot spot prediction in protein-protein interactions by an ensemble system
Source: BMC Syst Biol. 2018 Dec 31;12(Suppl 9):132. doi: 10.1186/s12918-018-0665-8 (PMC6311905; doi:10.1186/s12918-018-0665-8)
Supplement: Supplementary file 2 — All relevant MATLAB code. A simple Matlab implement of our predictor. (ZIP 19,742 kb) [file 12918_2018_665_MOESM2_ESM.zip › Additional file 2/Read ASA/ASA Property/BID/1K4UP.txt]

 NetSurfP - Protein Surface Accessibility and Secondary Structure Predictions

  

|  |  |  |
| --- | --- | --- |
|  |  | NetSurfP - Protein Surface Accessibility and Secondary Structure Predictions  Technical University of Denmark |

  


---

```
# For publication of results, please cite:
# A generic method for assignment of reliability scores applied to solvent accessibility predictions.
# Bent Petersen, Thomas Nordahl Petersen, Pernille Andersen, Morten Nielsen and Claus Lundegaard
# BMC Structural Biology 2009, 9:51 doi:10.1186/1472-6807-9-51
#
# Column 1: Class assignment - B for buried or E for Exposed - Threshold: 25% exposure, but not based on RSA
# Column 2: Amino acid
# Column 3: Sequence name
# Column 4: Amino acid number
# Column 5: Relative Surface Accessibility - RSA
# Column 6: Absolute Surface Accessibility
# Column 7: Z-fit score for RSA prediction
# Column 8: Probability for Alpha-Helix
# Column 9: Probability for Beta-strand
# Column 10: Probability for Coil
E S  1K4UP                  1    0.934 109.418  -0.004   0.003   0.003   0.994
E K  1K4UP                  2    0.708 145.594   0.302   0.018   0.019   0.964
E P  1K4UP                  3    0.530  75.250  -1.841   0.018   0.047   0.935
E Q  1K4UP                  4    0.589 105.178  -0.149   0.018   0.047   0.935
E P  1K4UP                  5    0.477  67.630  -1.293   0.018   0.047   0.935
E A  1K4UP                  6    0.518  57.051  -1.270   0.018   0.047   0.935
B V  1K4UP                  7    0.261  40.162  -0.224   0.018   0.047   0.935
E P  1K4UP                  8    0.366  51.964  -0.471   0.005   0.015   0.979
E P  1K4UP                  9    0.468  66.338  -1.453   0.018   0.019   0.964
E R  1K4UP                 10    0.465 106.462  -0.347   0.018   0.019   0.964
E P  1K4UP                 11    0.352  49.892  -1.131   0.058   0.017   0.925
E S  1K4UP                 12    0.491  57.592  -1.302   0.181   0.016   0.803
E A  1K4UP                 13    0.403  44.356  -1.011   0.430   0.016   0.555
E D  1K4UP                 14    0.476  68.620  -0.676   0.561   0.047   0.393
B L  1K4UP                 15    0.166  30.431   0.200   0.701   0.107   0.192
B I  1K4UP                 16    0.102  18.888  -0.444   0.701   0.107   0.192
B L  1K4UP                 17    0.165  30.193   0.149   0.751   0.050   0.199
E N  1K4UP                 18    0.449  65.734  -0.173   0.660   0.049   0.291
E R  1K4UP                 19    0.355  81.272   0.711   0.660   0.049   0.291
B C  1K4UP                 20    0.063   8.831  -0.354   0.430   0.016   0.555
E S  1K4UP                 21    0.397  46.493  -0.281   0.339   0.016   0.645
E E  1K4UP                 22    0.645 112.647  -0.055   0.802   0.014   0.185
E S  1K4UP                 23    0.468  54.861  -0.617   0.782   0.003   0.216
B T  1K4UP                 24    0.195  27.005  -0.928   0.858   0.002   0.139
E K  1K4UP                 25    0.441  90.734  -0.044   0.858   0.002   0.139
E R  1K4UP                 26    0.510 116.744   0.511   0.858   0.002   0.139
E K  1K4UP                 27    0.463  95.301   0.874   0.879   0.010   0.111
B L  1K4UP                 28    0.157  28.838  -0.929   0.782   0.003   0.216
E A  1K4UP                 29    0.470  51.827  -0.804   0.717   0.014   0.269
E S  1K4UP                 30    0.695  81.419  -0.312   0.522   0.016   0.462
E A  1K4UP                 31    0.500  55.100  -0.676   0.321   0.003   0.675
E V  1K4UP                 32    0.756 116.166  -1.279   0.016   0.005   0.979

Explain the output.  Go back.
```

---
